# Association between triglyceride-glucose index and subclinical carotid atherosclerosis in systemic lupus erythematosus: a cohort-based study

**DOI:** 10.3389/fimmu.2025.1614970

**Published:** 2025-06-25

**Authors:** Wenhui Xie, Minjie Li, Hong Huang, Yong Fan, Dai Gao, Jiaying Zhang, Zhuoli Zhang

**Affiliations:** Department of Rheumatology and Clinical Immunology, Peking University First Hospital, Beijing, China

**Keywords:** systemic lupus erythematosus, triglyceride-glucose index, carotid atherosclerosis, carotid artery plaque, insulin resistance

## Abstract

**Objective:**

To investigate the association between triglyceride-glucose (TyG) index and carotid atherosclerosis in patients with systemic lupus erythematosus (SLE).

**Methods:**

In this tertiary-center cross-sectional study, 333 consecutive SLE patients undergoing carotid ultrasonography were stratified by TyG index tertiles. The TyG index was calculated as ln[fasting triglycerides (mg/dL) × fasting glucose (mg/dL)/2]. Participants were categorized according to the TyG index tertiles: tertile 1 (<8.25), tertile 2 (8.25–8.69), and tertile 3 (>8.70). Multivariable logistic regression models adjusted for age, sex, body mass index (BMI), comorbidities, low-density lipoprotein cholesterol (LDL-c), statin, and SLE-specific covariates were employed to assess relationships between the TyG index and carotid atherosclerosis/plaque presence.

**Results:**

The overall rate of carotid atherosclerosis was 10.5% (35/333), with significantly elevated TyG in affected versus unaffected patients (8.77 ± 0.45 vs. 8.45 ± 0.49; p < 0.001). The frequency of carotid atherosclerosis was increased with increases in TyG tertiles (3.6% for tertile 1, 10.0% for tertile 2, and 17.7% for tertile 3; p = 0.003). Each 1-unit TyG increase was associated with a 4.29-fold increased atherosclerosis risk after full adjustment (95% CI 1.47–12.53). Compared to tertile 1, tertile 3 participants exhibited 5.58-fold increased odds of atherosclerosis (95% CI 1.52–20.53; p for trend 0.021). Consistent patterns were observed for carotid plaque outcomes.

**Conclusions:**

Elevated TyG index independently predicts carotid atherosclerosis risk in SLE populations beyond traditional cardiovascular and lupus-specific confounders. This accessible metabolic biomarker may enhance early atherosclerotic risk stratification in SLE management.

## Introduction

Systemic lupus erythematosus (SLE), a multisystem autoimmune disease predominantly affecting women of reproductive age, demonstrates a global prevalence of 43.7 per 100,000 individuals, affecting 3.41 million people worldwide (1). Extensive evidence shows that SLE patients confer substantially increased risks of cardiovascular complications, which have become the primary cause of premature mortality worldwide (2–4). Carotid ultrasonography has emerged as the gold standard for the non-invasive assessment of subclinical atherosclerosis through the detection of carotid artery plaque (CAP) and carotid intima–media thickness (cIMT) (5). Given that atherosclerotic changes precede clinical cardiovascular events by 5–7 years, the early detection of carotid atherosclerosis (CA) represents a critical preventive strategy in SLE management.

The triglyceride-glucose (TyG) index, calculated as ln[fasting triglycerides (mg/dL) × fasting blood glucose (mg/dL)/2], has gained recognition as a validated surrogate biomarker for insulin resistance since its initial proposition in 2008 (6). Cumulative evidence from population-based studies has demonstrated the predictive capacity of the TyG index for cardiovascular outcomes in the general population and specific populations of diabetes, coronary artery disease, and kidney transplant (7–10). The relationship between the TyG index and subclinical atherosclerosis was recently reported although inconclusive (11–13). SLE populations at high risk of premature cardiovascular diseases present unique atherogenic challenges distinct from the general population for chronic inflammation and metabolic disturbances due to the nature of the disease itself. In the SLE population, a previous cross-sectional study of 57 SLE patients showed that a higher TyG index was positively associated with hypertension, body mass index, waist circumference, fat mass, dyslipidemia, and uric acid levels (14). However, so far, there is no study on the relationship between the TyG index and cardiovascular abnormalities in SLE patients. Hence, we aimed to investigate the association between the TyG index and atherosclerotic risk among SLE patients.

## Methods

### Study design and patients

This is a cross-sectional study that utilized baseline data from January 2020 to December 2023 based on the Collaboratively Intensive Treat-to-target in the SLE cohort (STAR cohort), a longitudinal observational cohort. All patients met either the 1997 revised SLE American College of Rheumatology (ACR) classification criteria or the 2012 Systemic Lupus International Collaborating Clinics (SLICC) classification criteria. More details of the cohort have been previously described (15, 16). The study was approved by the institutional review board of the Peking University First Hospital (project 2017 (1284)). Written informed consent was obtained from all participants. All the procedures were performed in accordance with the Declaration of Helsinki of 1964 and its later amendments or comparable ethical standards.

The inclusion criteria were as follows: 1) age ≥ 18 years at enrollment, 2) complete carotid ultrasonography, and 3) fasting triglycerides and blood glucose concentrations at the same time of carotid artery ultrasound examination. The exclusion criteria were as follows: 1) comorbid systemic autoimmune disorders (e.g., rheumatoid arthritis), 2) pre-existing overt cardiovascular diagnoses, and 3) current use of antidiabetic agents (e.g., insulin and oral hypoglycemics) or triglyceride-lowering drugs (e.g., fibrates and icosapent ethyl).

### Clinical assessments

The data recorded through face-to-face interviews for eligible participants included i) demographics and cardiometabolic profile: age, gender, education, body mass index (BMI), smoking status, drinking, comorbidities (including hypertension, hypercholesterolemia, hyperuricemia, and hyperhomocysteinemia), total cholesterol (TCHO), low-density lipoprotein cholesterol (LDL-c), uric acid, and homocysteine; ii) SLE-specific parameters: family history of connective tissue disease (CTD), disease duration, the Systemic Lupus Erythematosus Disease Activity Index 2000 (SLEDAI-2K), physician’s global assessment (PGA; range 0–3.0), the SLICC/ACR Damage Index (SDI), anti-dsDNA antibody positivity, complement 3/4, hypocomplementemia, serum creatinine, secondary antiphospholipid syndrome, and SLE treatment; and iii) TyG and carotid ultrasound variables: fasting triglycerides, fasting blood glucose concentrations, and carotid artery ultrasound data.

All biochemical/immunological assays were performed in the Clinical Lab of Peking University First Hospital, which has a laboratory accreditation certificate. Glucose and triglyceride values underwent standardized unit conversion by multiplying by 18.020 and 88.545, respectively. The TyG index was computed as ln[triglyceride (mg/dL) × fasting glucose (mg/dL)/2] (6). Cigarette smoking was defined as smoking at least one cigarette per day for more than 1 year. Alcohol intake was defined as the consumption of at least 30 g of alcohol per week for at least 1 year. BMI was calculated as weight (kg)/height (m)^2^. All the comorbidities were assessed according to the corresponding criteria. SLEDAI-2K, PGA, and SDI were adopted during evaluation by trained rheumatologists, as described in our previous study (15, 16). SLEDAI-2K score was adopted to reflect the disease activity, with 0–4 as inactive and ≥5 as active.

### Carotid artery measurements

Carotid ultrasonography of bilateral carotid arteries was conducted by two certified sonographers with unified training. They were blinded to the clinical characteristics and laboratory results of the subjects. Discordant outcomes were resolved through consensus or consultation. A patient with increased cIMT or CAP was defined as having CA. Increased cIMT was defined as cIMT ≥ 0.9 mm in either the left or right carotid artery. CAP was defined as cIMT ≥ 1.5 mm, a focal structure encroaching into the arterial lumen of at least 0.5 mm, or cIMT ≥ 50% of the surrounding cIMT value. The primary outcome assessed the cross-sectional association between TyG index tertiles and CA prevalence, with CAP presence analyzed as a secondary endpoint.

### Statistical analysis

Continuous variables are shown as the mean ± standard deviation (SD) or median with interquartile ranges (IQRs), and categorical variables are expressed as absolute frequencies (proportions). The TyG index was analyzed as both continuous (per 1-SD increment) and ordinal tertile variables. The participants were classified into three groups according to the tertile level of the TyG index. Comparisons among groups were performed using the one-way analysis of variance or the Kruskal–Wallis test for continuous variables, depending on normality, while the chi-square was used for categorical variables. The odds ratio (OR) with a 95% confidence interval (CI) was used to calculate the association between the TyG index and CA. Four hierarchical logistic regression models were conducted in the present study: (Model 1) crude; (Model 2) adjusted for sex and age; (Model 3) adjusted for sex, age, BMI, comorbidities, and LDL-c; and (Model 4) adjusted for sex, age, BMI, comorbidities, statin, and LDL-c as well as SLE-related variables (SLEDAI-2K, PGA, lupus nephritis, and treatment). Furthermore, subgroup analyses were performed between different characteristics (age, gender, smoking history, etc.). The median value for continuous variables (e.g., age) was applied in the subgroup analyses. The interactions between the TyG index and covariates of interest were tested using the likelihood ratio test of models with interaction terms. All analyses were performed with Stata 13.1 (STATA Corp.). Microsoft Excel 2010 and GraphPad Prism 8.0 (San Diego, CA, USA) were used to produce the graphs. Statistical significance was defined as two-tailed p < 0.05.

## Results

### Characteristics of the study participants

**Figure 1:**
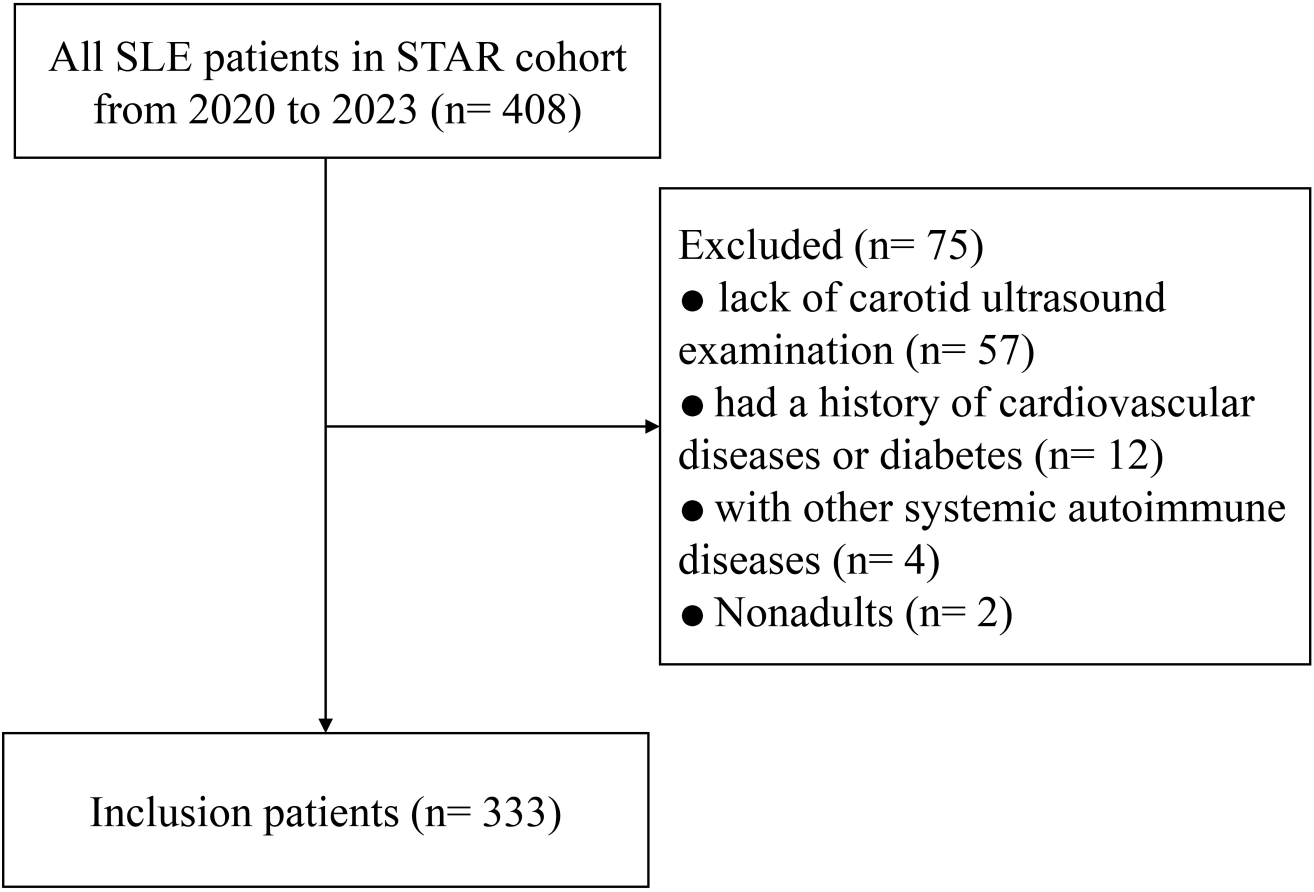
Study flowchart of the inclusion of study participants.

**Figure 2:**
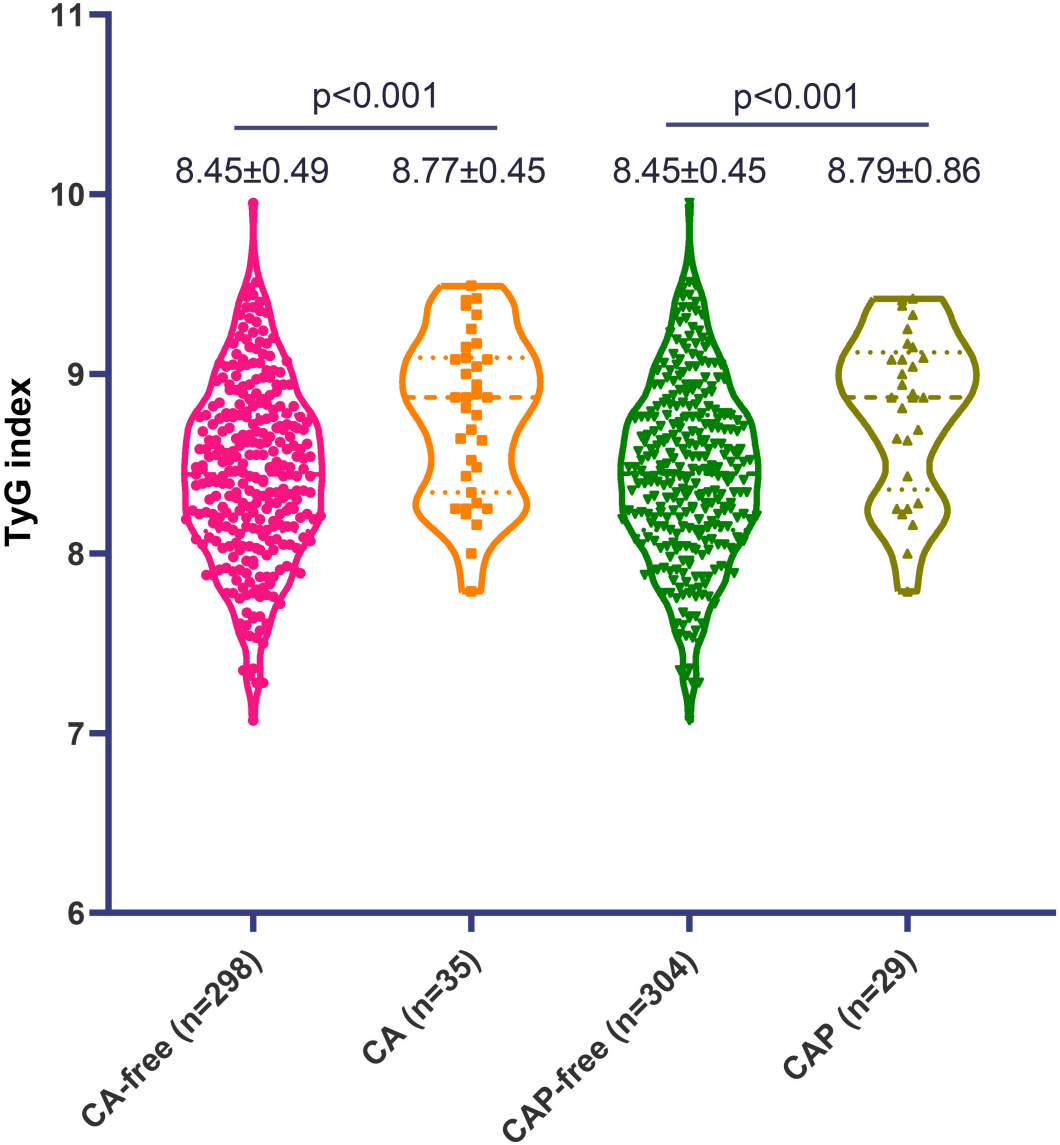
The level of triglyceride-glucose (TyG) index in patients with systemic lupus erythematosus, stratified by carotid atherosclerosis (CA) and carotid artery plaque (CAP).

Finally, a total of 333 consecutive SLE participants were eligible according to the inclusion criteria. The patient inclusion flowchart is presented in **Figure 1**. The median disease duration was 94 months, the mean age was 39.1 (12.9) years, and 304 (91.3%) patients were female. The mean (SD) TyG index of the individuals was 8.49 (0.54). There were 35 (10.5%) and 29 (8.7%) patients identified as having CA and CAP, respectively. Overall, SLE patients with either CA or CAP had a substantially higher TyG index compared with those without (8.77 ± 0.45 vs. 8.45 ± 0.49, p < 0.001; or 8.79 ± 0.86 vs. 8.45 ± 0.45, p = 0.001, respectively) (**Figure 2**).

Participants were stratified into three groups according to the tertiles of the TyG index level (tertile 1, n = 110, <8.25; tertile 2, n = 110, 8.25–8.69; and tertile 3, n = 113, ≥8.70). The mean (SD) TyG index was 7.94 (0.25), 8.47 (0.13), and 9.04 (0.24) in patients in tertiles 1, 2, and 3, respectively (**Table 1**). Demographics and clinical and laboratory characteristics according to the tertiles of the TyG index are presented in **Table 1**. In general, the levels of BMI, uric acid, homocysteine, TCHO and LDL-c, fasting plasma glucose, and fasting triglyceride were all positively correlated with increasing TyG index tertiles. Patients in tertiles 2 and 3 of the TyG index were more likely to have a shorter disease duration and higher SLEDAI-2K and PGA scores, compared to those in tertile 1. In terms of pharmacological treatment, compared to the tertile 1 group, patients in the tertile 2 and 3 groups– exhibited a higher proportion of pulse steroid therapy administration, higher current steroid dosage levels, and increased utilization rate of immunosuppressants (**Table 1**).

**Table 1:** Demographics and clinical characteristics of patients with systemic lupus erythematosus, stratified by the tertiles of TyG index.

	Total	TyG index tertiles			p-Value
		Tertile 1 (<8.25; n = 110)	Tertile 2 (8.25–8.69; n = 110)	Tertile 3 (≥8.70; n = 113)	
Demographics					
Age at visit, years	39.1 ± 12.9	38.0 ± 11.0	39.3 ± 13.9	40.1 ± 13.7	0.468
Female sex, n (%)	304 (91.3)	100 (90.9)	99 (90.0)	105 (92.9)	0.221
Cigarette smoking, n (%)	27/330 (8.2)	6/109 (5.5)	9/109 (8.3)	12/112 (10.7)	0.368
Drinking, n (%)	16/255 (6.3)	9/80 (11.3)	4/84 (4.8)	3/90 (3.3)	0.204
Body mass index, kg/m^2^	23.1 ± 4.5	22.9 ± 5.1	22.4 ± 3.6	24.0 ± 4.6	0.018
Higher education, n (%)	196/257 (76.3)	69/82 (84.1)	59/84 (70.2)	68/91 (74.7)	0.099
Hypertension, n (%)	28 (8.4)	6 (5.5)	12 (10.9)	10 (8.8)	0.338
Hypercholesterolemia, n (%)	39 (11.7)	3 (2.7)	11 (10.0)	25 (22.1)	<0.001
Hyperuricemia, n (%)	43 (12.9)	11 (10.0)	8 (7.3)	24 (21.2)	0.004
Hyperhomocysteinemia, n (%)	69/264 (26.1)	19/85 (22.4)	17/86 (19.8)	33/93 (35.5)	0.036
Total cholesterol, mmol/L	4.5 ± 1.1	4.2 ± 0.7	4.4 ± 1.0	4.9 ± 1.4	<0.001
LDL cholesterol, mmol/L	2.5 ± 0.9	2.3 ± 0.6	2.5 ± 0.7	2.8 ± 1.1	<0.001
Uric acid, μmol/L	325.2 ± 90.0	312.2 ± 79.0	311.4 ± 79.8	351.0 ± 103.3	0.001
Homocysteine, μmol/L	13.9 ± 6.7	13.5 ± 6.3	12.7 ± 4.3	15.3 ± 8.4	0.025
Disease features					
Disease duration, months	94 (36–170)	117 (53–190)	89 (32–161)	89 (25–160)	0.035
Family history of CTD, n (%)	36/265 (13.6)	16/83 (19.3)	11/88 (12.5)	9/93 (9.7)	0.167
PGA, 0–3	0.5 (0.0–1.0)	0.3 (0.0–0.6)	0.5 (0.0–1.0)	1.0 (0.0–1.5)	<0.001
PGA > 0.5, n (%)	130 (39.0)	27 (24.5)	41 (37.3)	62 (54.9)	<0.001
SLEDAI-2K, 0–105	3.0 (1.0–6.0)	2.0 (0.0–4.0)	2.5 (0.8–6.0)	4.0 (2.0–10.0)	<0.001
SLEDAI-2K > 4, n (%)	172 (51.7)	45 (40.9)	56 (50.9)	71 (62.8)	0.005
SDI, 0–47	0.0 (0.0–1.0)	0.0 (0.0–1.0)	0.0 (0.0–1.0)	0.0 (0.0–1.0)	0.127
SDI > 0, n (%)	88/268 (32.8)	22/87 (25.3)	31/92 (33.7)	35/89 (39.3)	0.137
Anti-dsDNA positivity, n (%)	198 (59.5)	62 (56.4)	60 (54.5)	76 (67.3)	0.699
Hypocomplementemia, n (%)	135 (40.5)	42 (38.2)	48 (43.6)	45 (39.8)	0.699
Complement 3, g/L	0.71 (0.58–0.82)	0.72 (0.59–0.79)	0.68 (0.56–0.82)	0.73 (0.58–0.89)	0.413
Complement 4, g/L	0.15 (0.11–0.19)	0.14 (0.11–0.19)	0.15 (0.11–0.20)	0.15 (0.11–0.19)	0.829
Serum creatinine, μmol/L	69.0 (62.0–77.0)	70.0 (64.0–77.3)	67.0 (62.0–76.3)	68.7 (62.0–80.3)	0.278
Lupus nephritis	161 (48.3)	48 (43.7)	60 (54.5)	53 (46.9)	0.251
Secondary APS	25 (7.5)	4 (3.6)	10 (9.1)	11 (9.7)	0.167
Current glucocorticoid dosages	5.00 (1.25–10.00)	5.00 (0–10.00)	6.25 (1.25–15.00)	8.75 (1.25–40.0)	0.100
Pulse steroid therapy, ever, n (%)	16 (4.8)	2 (1.8)	4 (3.6)	10 (8.8)	0.038
Immunosuppressant use, n (%)	217 (65.2)	59 (53.6)	81 (73.6)	77 (68.1)	0.006
Hydroxychloroquine, n (%)	306 (91.9)	105 (95.5)	98 (89.1)	103 (91.2)	0.211
Statins	26 (7.8)	3 (2.7)	8 (3.8)	15 (13.3)	0.013
TyG variables					
Fasting plasma glucose, mmol/L	4.85 ± 0.64	4.67 ± 0.52	4.77 ± 0.53	4.95 ± 0.77	<0.001
Fasting triglyceride, mmol/L	1.40 ± 0.68	0.79 ± 0.20	1.28 ± 0.22	2.10 ± 0.61	<0.001
TyG index	8.48 ± 0.49	7.94 ± 0.25	8.47 ± 0.13	9.02 ± 0.24	<0.001

CTD, connective tissue disease; SLEDAI-2K, Systemic Lupus Erythematosus Disease Activity Index 2000; SDI, the Systemic Lupus International Collaborating Clinics/American College of Rheumatology Damage Index; PGA, physician’s global assessment; APS, antiphospholipid Syndrome; TyG, triglyceride-glucose.

**Figure 3:**
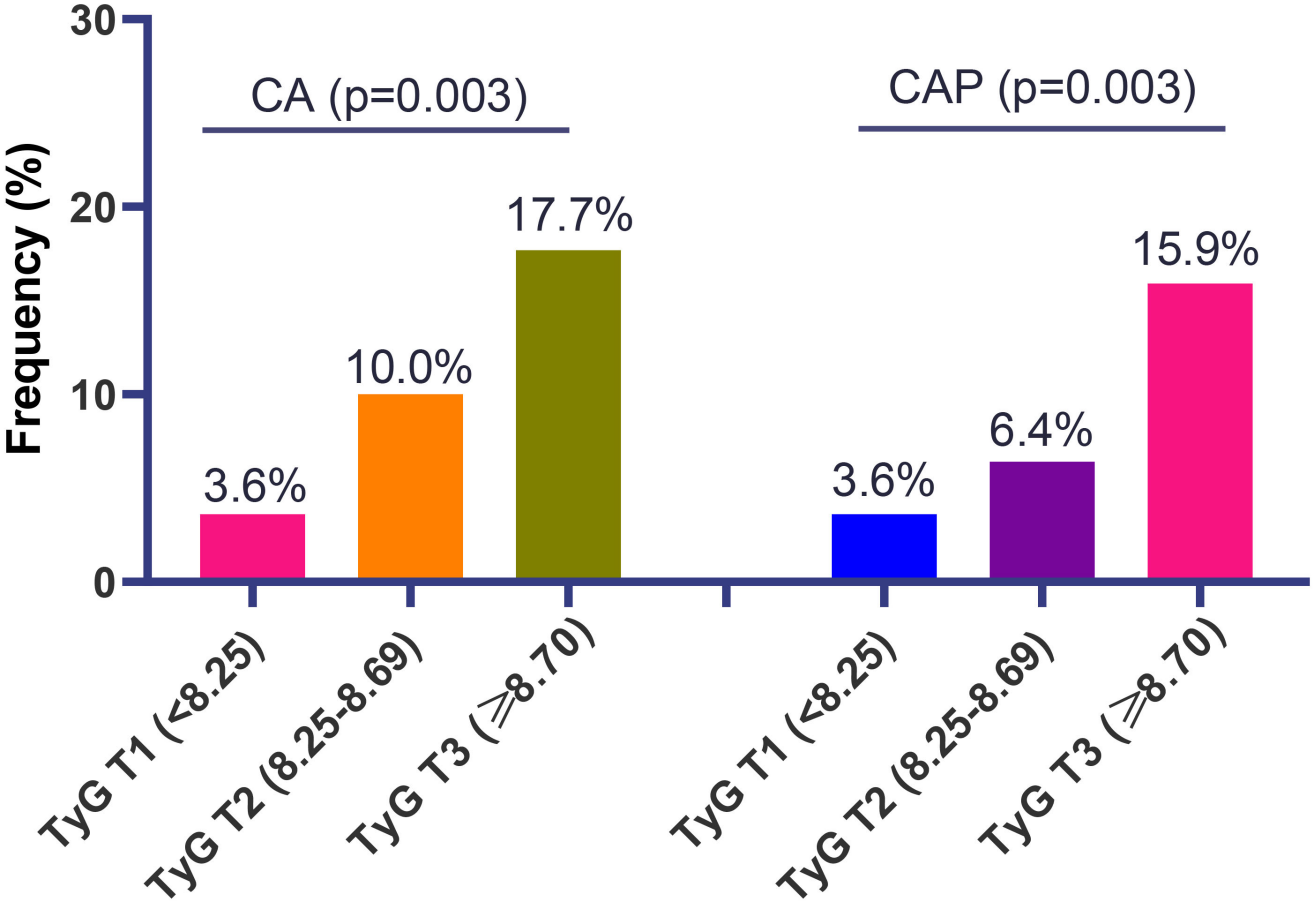
The frequency of carotid atherosclerosis (CA) and carotid artery plaque (CAP) in patients with systemic lupus erythematosus, according to different tertiles of triglyceride-glucose (TyG) index.

For the atherosclerotic findings, the higher frequency of CA was observed with increasing tertiles of the TyG index, yielding 3.6% (4/110) for tertile 1, 10.0% (11/110) for tertile 2, and 17.7% (20/113) for tertile 3 (p = 0.003). Similar findings were noted for the CAP as the outcome (3.6% for tertile 1, 6.4% for tertile 2, and 15.9% for tertile 3; p = 0.003) (**Figure 3**).

### Association of TyG index with CA and CAP

The results of logistic regression analysis on the associations of the TyG index with CA and CAP are presented in **Table 2**. When the TyG index was used as a continuous variable, the crude OR values of CA and CAP were elevated by 307% (OR 4.07, 95% CI 1.87–8.87) and 351% (OR 4.51, 95% CI 1.93–10.55) for every 1-unit increase in the TyG index. In subsequent adjusted models, significant associations between continuous TyG and CA and CAP were noted, with fully adjusted ORs of 4.29 (95% CI 1.47–12.53) and 4.59 (95% CI 1.42–14.81), respectively.

**Table 2:** Odds ratios for association of the TyG index with carotid atherosclerosis and carotid artery plaque in patients with systemic lupus erythematosus.

	Each 1-unit increase	p-Value	TyG index tertiles			p for trend
			Tertile 1 (<8.25; n = 110)	Tertile 2 (8.25–8.69; n = 110)	Tertile 3 (≥8.70; n = 113)	
Carotid atherosclerosis						
Model 1	4.07 (1.87–8.87)	<0.001	Ref.	2.94 (0.91–9.55)	5.70 (1.88–17.28)	0.006
Model 2	4.09 (1.66–10.05)	0.002	Ref.	2.38 (0.68–8.36)	4.96 (1.53–16.03)	0.020
Model 3	4.33 (1.61–11.64)	0.004	Ref.	2.31 (0.63–8.57)	5.18 (1.49–17.99)	0.027
Model 4	4.29 (1.47–12.53)	0.008	Ref.	2.07 (0.53–8.07)	5.58 (1.52–20.53)	0.021
Carotid artery plaque						
Model 1	4.51 (1.93–10.55)	0.001	Ref.	1.80 (0.51–6.34)	5.02 (1.64–15.36)	0.006
Model 2	4.45 (1.66–11.93)	0.003	Ref.	1.29 (0.33–5.04)	4.16 (1.26–13.68)	0.019
Model 3	4.25 (1.42–12.70)	0.010	Ref.	1.25 (0.30–5.31)	4.16 (1.14–15.19)	0.039
Model 4	4.59 (1.42–14.81)	0.011	Ref.	1.25 (0.28–5.60)	4.88 (1.27–18.67)	0.024

TyG, triglyceride-glucose; BMI, body mass index; LDL-c, low-density lipoprotein cholesterol; SLE, systemic lupus erythematosus; SLEDAI-2K, Systemic Lupus Erythematosus Disease Activity Index 2000; PGA, physician’s global assessment.

Model 1: unadjusted. Model 2: adjusted for age and sex. Model 3: adjusted for age, sex, comorbidities, BMI, LDL-c, and statins. Model 4: adjusted for age, sex, comorbidities, BMI, LDL-c, statins, and SLE-related variables (SLEDAI-2K, PGA, lupus nephritis, and treatment).

Compared with the lowest tertile of the TyG index, tertile 3 was significantly associated with a higher prevalence of CA (OR 4.96, 95% CI 1.53–16.03, p for trend 0.020) and CAP (OR 4.16, 95% CI 1.26–13.68, p for trend 0.019) in the age- and gender-adjusted logistic regression models (**Table 2**). After fully adjusting for all covariables in Model 4, higher TyG tertiles remained significantly associated with an increased frequency of CA (p for trend 0.021) and CAP (p for trend 0.024), with ORs (95% CIs) for tertile 3 versus tertile 1 of 5.58 (1.52–20.53) and 4.88 (1.27–18.67), respectively.

### Subgroup analyses

To investigate the presence of subgroup differences in terms of the association between a continuous TyG index and prevalence of CA or CAP, we conducted subgroup analyses according to the potential atherosclerosis risk factors, including age (<37 vs. ≥37 years), gender (female vs. male), smoking history (yes vs. no), drinking history (yes vs. no), BMI (<25 vs. ≥25 kg/m^2^), hypertension (yes vs. no), hypercholesterolemia (yes vs. no), hyperuricemia (yes vs. no), hyperhomocysteinemia (yes vs. no), disease duration of SLE (<94.2 vs. ≥94.2 months), family history of CTD (yes vs. no), PGA score (≤0.5 vs. >0.5), SLEDAI-2K score (≤4 vs. >4), lupus nephritis (yes vs. no), anti-dsDNA positivity (yes vs. no), and hypocomplementemia (yes vs. no) (**Table 3**). Overall, there were no significant interactions detected in all categories (p for interaction >0.05).

**Table 3:** Subgroup analyses for the association of the continuous TyG index with carotid atherosclerosis and carotid artery plaque in patients with systemic lupus erythematosus.

Subgroup	*Carotid atherosclerosis*		*Carotid artery plaque*	
	OR (95% CI)	p _interaction_	OR (95% CI)	p _interaction_
Age				
<37 years	6.40 (0.07–553.19)	0.186	6.40 (0.07–553.19)	0.148
≥37 years	4.30 (1.79–10.35)		4.66 (1.81–11.98)	
Gender				
Female	4.03 (1.83–8.91)	0.919	4.51 (1.91–10.66)	NA
Male	4.17 (0.10–175.88)		NA	
Education				
No	16.10 (1.86–139.34)	0.093	10.04 (1.13–89.13)	0.136
Yes	2.52 (0.92–6.89)		3.58 (1.12–11.49)	
Smoking history				
No	3.40 (1.37–8.40)	0.304	3.93 (1.43–10.83)	0.278
Yes	2.74 (0.28–27.18)		9.15 (0.20–412.91)	
Drinking history				
No	4.38 (1.67–11.47)	0.812	5.20 (1.79–15.10)	NA
Yes	4.48 (0.16–122.47)		NA	
BMI				
<25 kg/m^2^	5.38 (2.00–14.45)	0.792	6.20 (2.00–19.19)	0.956
≥25 kg/m^2^	2.64 (0.68–10.21)		2.64 (0.68–10.21)	
History of hypertension				
No	5.54 (2.20–13.95)	0.687	6.59 (2.34–18.56)	0.599
Yes	1.71 (0.27–11.10)		1.86 (0.27–12.79)	
History of hypercholesterolemia				
No	4.78 (2.06–11.13)	0.555	5.40 (2.11–13.81)	0.389
Yes	0.87 (0.83–9.00)		0.87 (0.83–9.00)	
History of hyperuricemia				
No	4.33 (1.90–9.89)	0.756	4.80 (1.93–11.96)	0.575
Yes	2.61 (0.25–27.68)		2.61 (0.25–27.68)	
History of hyperhomocysteinemia				
No	4.52 (1.61–12.68)	0.304	5.62 (1.70–18.63)	0.600
Yes	4.39 (0.84–22.91)		4.39 (0.84–22.91)	
Disease duration				
<94.2 months	3.51 (1.20–10.26)	0.366	4.12 (1.20–14.08)	0.337
≥94.2 months	4.75 (1.52–14.91)		5.10 (1.56–16.72)	
Family history of CTD				
No	1.77 (1.77–12.85)	0.378	6.73 (2.06–21.94)	0.460
Yes	1.58 (0.20–12.50)		1.58 (0.20–12.50)	
PGA score				
≤0.5	6.61 (2.28–19.10)	0.876	8.26 (2.60–26.29)	0.670
>0.5	2.81 (0.77–10.28)		2.78 (0.65–11.88)	
SLEDAI-2K score				
≤4	3.62 (1.31–10.00)	0.884	4.07 (1.40–11.86)	0.623
>4	5.85 (1.64–20.96)		7.38 (1.64–33.17)	
Lupus nephritis				
No	4.96 (1.16–20.559)	0.835	7.12 (1.29–39.27)	0.724
Yes	3.95 (1.15–13.62)		3.79 (1.02–14.00)	
Anti-dsDNA positivity				
No	3.04 (1.04–8.85)	0.827	3.75 (1.16–12.14)	0.781
Yes	5.98 (1.87–19.08)		5.83 (1.66–20.40)	
Hypocomplementemia				
No	4.90 (1.93–12.45)	0.332	5.70 (2.10–15.47)	0.780
Yes	2.55 (0.590–11.03)		2.13 (0.37–12.23)	

BMI, body mass index; CTD, connective tissue disease; PGA, physician’s global assessment; SLEDAI-2K, Systemic Lupus Erythematosus Disease Activity Index 2000.

## Discussion

In this cross-sectional study, we first explored the relationship between the TyG index and CA among the SLE population. A higher TyG index was significantly associated with a greater likelihood of CA and CAP in both continuous and tertile forms. Similar findings were validated in all the subgroup analyses, indicating the robustness of the associations. Our study uncovered that the TyG index may be a useful marker for subclinical atherosclerosis among patients with SLE and may become a simple, convenient, and effective tool for cardiovascular risk assessment in daily practice.

Large epidemiological studies have proven that cardiovascular burden is significantly increased in individuals with SLE, mainly caused by traditional cardiovascular risk and chronic systemic inflammation (2–4). In consideration of CA being an important process of clinically overt cardiovascular diseases, the early identification and intervention of CA are therefore essential for better cardiovascular outcomes (5). The TyG index, recognized as an insulin resistance marker, has been extensively studied in the field of atherosclerotic cardiovascular disease. Extensive literature has suggested that the TyG index is independently associated with the incidence, development, and adverse outcomes of cardiovascular complications, mostly in the general population (7–13). In the SLE population, a previous study of 57 SLE patients showed that a higher TyG index was associated with hypertension, BMI, waist circumference, fat mass, dyslipidemia, and uric acid level (14). At present, no study has reported the relation of the TyG index with cardiovascular abnormalities among the SLE population. In our cross-sectional study, a significant relationship between the TyG index and subclinical atherosclerosis among SLE patients has been verified for the first time. On the one hand, the association was confirmed even after correction for other confounding factors related to cardiovascular risk factors and related SLE. The independent and robust association indicated that the TyG index may be a promising atherosclerotic marker for the early identification of SLE patients at high risk of atherosclerotic events. On the other hand, the robustness and consistency of the association across different subgroups were also supported by all subgroup analyses. This study demonstrated that each 1-unit TyG index increase confers approximately fivefold higher atherosclerosis risk in SLE patients after full adjustment, which seems to be greater than the 1.5-fold risk observed in general populations (11). Meanwhile, the mean TyG in SLE patients appears to be higher than that in the general Chinese population, especially in consideration of age and sex distribution in the SLE population (17, 18). On the one hand, SLE patients generally confer heightened cardiometabolic risk and more severe atherosclerotic disease compared with both general populations because of the higher systemic inflammatory burden (19). On the other hand, systemic inflammation is also caused by unique immune dysregulation featuring type I interferon excess, aberrant leukocyte activation, and autoantibody-mediated vascular injury (20). Moreover, specific organ involvement in SLE, particularly renal disease, accelerates atherosclerosis progression. As demonstrated by Fayed et al. (21), patients with lupus nephritis exhibit more severe endothelial impairment compared to non-LN SLE patients and healthy controls, highlighting the synergistic impact of autoimmunity and renal injury on vascular health. These processes synergistically promote insulin resistance and endothelial dysfunction, rendering TyG a more sensitive atherosclerosis indicator in SLE. Nevertheless, the wide confidence intervals of estimates in our study warrant validation through multicenter prospective studies with larger event cohorts.

The exact mechanisms underlying the association between the TyG index and cardiovascular complications have not been exactly clarified. Possible explanations are as follows. First, the TyG index, being a reliable marker of insulin resistance, may be one of the explanations for this association (22). Insulin resistance has been identified as a clear risk factor for cardiovascular diseases (23). On the one hand, insulin resistance can induce inflammation and endothelial dysfunction via glucose metabolism imbalance and hyperglycemia (24). On the other hand, insulin resistance also can cause dysfunction of vascular contraction and relaxation through the increased production of free radicals and nitric oxide inactivation (25). These contribute to atherosclerotic plaque formation and the development of cardiovascular complications. In addition, our results showed that a higher tertile of the TyG index was associated with a wide range of traditional cardiovascular risk factors, including higher BMI, metabolic disorders, and SLE-related risk factors. These factors may contribute to the initiation and acceleration of atherosclerosis (26). Moreover, increased triglyceride levels can cause increased free fatty acid levels, further promote endothelial dysfunction, and exert an atherogenic effect on atherosclerosis via triglyceride-rich lipoproteins (27, 28). Meanwhile, hyperglycemia also could accelerate atherosclerosis by inducing mitochondrial ROS overproduction, which drives advanced glycation end-product formation, protein kinase C activation, and increased polyol pathway flux (29).

We acknowledge that limitations exist in the present study. First, a causal relationship cannot be established based on the cross-sectional study design and relatively limited events. TyG index level measurement at a single timepoint may not fully reflect long-term metabolic exposure related to plaque formation. Future prospective studies employing large samples with consideration of dynamic change of TyG are required to prove the association. Second, the inherent variability of single-timepoint measurements of triglycerides and blood glucose may compromise the precision of TyG for individual risk stratification. Future studies could explore more stable biomarkers; hemoglobin A1 represents one such candidate as an established marker for chronic glycemic exposure. For instance, a previous investigation demonstrated that hemoglobin A1 independently correlated with both the presence and severity of coronary atherosclerosis in non-diabetic subjects, even under tightly controlled LDL-c conditions (30). Third, the nature of monocentric design limits the external validity and ability to generalize results to different regional and racial groups. The external validity and ability of the association of the TyG index with CA requires further prospective large-scale research. Fourth, the possibility of residual confounder (e.g., physical activity and diet) still cannot be excluded, although a wide range of potential atherosclerosis risk factors had been adjusted. In addition, several SLE-specific medications may also modulate atherosclerosis risk in SLE. For example, cumulative glucocorticoid dose was significantly associated with increased cIMT and carotid plaque (31), while hydroxychloroquine exerts atheroprotective effects by enhancing cholesterol efflux capacity, thereby elevating functional HDL levels (32). Last, subclinical atherosclerosis was exclusively focused on in this study; the value of the TyG index in clinically overt cardiovascular diseases should be investigated in the future.

## Conclusion

Our study demonstrated that elevated TyG index is independently associated with carotid atherosclerosis in SLE, supporting its clinical utility as a metabolic biomarker for cardiovascular risk stratification in lupus management in daily practice. Based on our findings, carotid ultrasound screening should be prioritized in SLE patients with a TyG index > 8.70.

## Data Availability

The raw data supporting the conclusions of this article will be made available by the authors, without undue reservation.
